# The Position of the Provincial Medical Schools

**Published:** 1891-09-05

**Authors:** 


### The Hospital

A Weekly Journal of

### Science, Medicine, Nursing, and Philanthropy.

*For Hospitals, Asylums, Infirmaries, Dispensaries, Medical Practitioners, Students, Nurses, and the Charitable Public.*

Vol. X.—No. 258.] [Sept. 5, 1891.

### The Position of the Provincial Medical Schools.

Amongst the many changes that have taken place in the medical profession during the last two generations, not the least noteworthy has been the growth in size and importance of the provincial medical schools. Fifty to sixty years ago, when they were comparatively small and obscure, few would have ventured to predict a successful future for them. They were then overshadowed, on the one hand, by the great London schools, with their prestige derived from the eminence of their teachers, past and present, their history, and material resources; and, on the other, by the flourishing Universities of Edinburgh and Dublin, which, in addition to their high reputation as medical schools, possessed the further advantage of being able to confer a degree. Indeed, there seemed every prospect that the medical education of the future would in Great Britain be entirely monopolised by these three great centres, and that the provincial schools would cease to exist. But about this time there began to be apparent that tendency of the population to accumulate in the great cities at the expense of the country districts and small towns, which has been such a remarkable feature in the England of this century, and with the rapid growth of these cities the position of the provincial medical schools was profoundly changed. The increased size of the towns provided an ever-increasing supply of clinical material which had at first been lacking, and the charity of the wealthier inhabitants in establishing large hospitals rendered that material accessible for the purposes of clinical instruction of students. With the growing prosperity of the large towns, there was developed in many places a local spirit which led the citizens to take pride in the efficiency of their institutions, and to contribute towards their support, whilst the greater interest that was now taken in educational matters led to the establishment of University Colleges for higher education, and in connection with these colleges the local Medical Schools received a fresh impulse.

The wisdom of judicious decentralisation is now so generally recognised that it is not necessary for us to do more than allude to the importance of this principle in medical matters. It is essential for the welfare of the whole community that all the medical talent of the country should not be concentrated in one place, and that our great cities should have capable men at the head of the local profession, and that there should be a sufficient inducement offered to clever men to stay in them. In what way this is to be secured without the stimulus that teaching affords and the opportunities for original investigation that the presence of a medical school supplies we are not able to imagine.

The conspicuous success of Cambridge University in developing its medical side as a centre for scientific education, and especially for the preliminary scientific training of medical students, was an encouragement to the other provincial medical schools to persevere in their efforts. They steadily improved their position by adding to their accommodation and their general arrangements for teaching, both in the direction of improved buildings and of a larger staff of teachers, until it may be said that at the present time they stand in no measure behind the London medical schools either in general efficiency or in success in turning out well-trained medical practitioners. When we call to mind the most distinguished names in the profession to-day we find that a by no means small proportion of those who have risen to eminence have at one time or another, either as students or as teachers, been connected with a provincial school of medicine.

As a sign of their activity within the last few years—to make no mention of the magnificent biological and physiological laboratories of Cambridge University, which stands on a different footing—new medical schools fitted up with all the most approved appliances have been erected at Newcastle, Leeds, Sheffield, Manchester, and at Birmingham.

Bristol, which does not possess the enormous wealth of the large manufacturing centres of the north, is at present engaged in building a large Medical School, which will give the requisite accommodation, and be completed in 1892. The building of the new school is due mainly to the energetic efforts made by the present teaching staff, and to the cordial support which they have received from the medical profession of the neighbourhood. They have also secured liberal aid from some of the more enlightened citizens who are interested in the cause of medical education, but they have not met with that widespread interest and help which they hoped for when they called attention to the indispensable service which the medical school renders in carrying on the large charitable institutions of the place. We think that the public have a very inadequate idea, for the most part, perhaps, no idea, of how essential the presence of medical students is for the efficient working of a large hospital, and what a large amount of routine work is done by them, work which, without their aid, would be largely left undone, or, if done, would entail additional administrative expenses.

Bristol, in point of foundation, is one of the oldest provincial schools, and has had many eminent men connected with it. It is affiliated to the University College, which has given generous aid to the scheme of the medical faculty. Unfortunately, the University College itself has languished for lack of funds, and from various causes has not received the support that the very able staff of teachers engaged in its work had a right to expect. We are afraid that the Bristol people have not shown that interest in the higher education of their sons which has been such a distinguishing characteristic of the great northern towns; but we hope that they may yet be stirred to give more aid to their Medical School and University College than they have hitherto done. In no way is the degree of enlightenment which a community has reached more manifest, and their freedom from provinciality better shown than in the encouragement they afford to higher education; and to take the lowest point of view, there is probably under modern conditions no form of expenditure which will yield in the future a richer harvest.

When the new Bristol medical school is built and equipped, a further need will come in, and that is, that its students should be able to obtain a medical degree without undue difficulty. We must now recognise that the Albert University, as it is to be constituted, will provide a degree for London students alone. The needs of the group of colleges in the North of England have been met in a similar manner by the establishment of the Victoria University; so that the Albert, Victoria, and Durham Universities provide a degree for the students of the constituent colleges which are situated in their districts respectively. At present it seems that Birmingham and Bristol will be left out in the cold. We cannot think, however, that this will be for long. Birmingham as the capital of the Midlands, and Bristol as the natural centre of the West of England, and as a large manufacturing town and seaport, together with the University College at Cardiff, as the centre for the large industrial districts of South Wales, are admirably situated to form the constituent colleges of a Midland or Midland and Western University on the same plan as the Victoria, at which their students will be able to obtain those advantages which are at present withheld from them, and to which they can put forward an undeniable claim. If we were asked to sum up briefly the advantages of a provincial medical school to the student, we should say that from the smaller number of students he is able to obtain more clinical experience, and, therefore, to gain a better practical knowledge of his work, and for the same reason he receives more individual attention from his teachers than is possible in the very large schools. Parents are also enabled to keep their boys at home, and thus to save expense, and at the same time to keep them under their own control at a very critical age for their moral welfare.

